# Facial magnetomyography using an array of optically pumped magnetometers

**DOI:** 10.1016/j.cnp.2025.03.003

**Published:** 2025-03-17

**Authors:** Johannes von Fraunberg, Hongyu Lu, Haodi Yang, Nura Marquetand, Christoph Braun, Lukas Rüttiger, Stephan Wolpert, Marlies Knipper, Markus Siegel, Hubert Löwenheim, Justus Marquetand

**Affiliations:** a Department of Otolaryngology, Head and Neck Surgery, Tübingen Hearing Research Centre, Molecular Physiology of Hearing, University of Tübingen, Tübingen, Germany; b Department of Neural Dynamics and Magnetoencephalography, Hertie-Institute for Clinical Brain Research, University of Tübingen, Tübingen, Germany; c MEG-Center, University of Tübingen, Tübingen, Germany; d Center for Integrative Neuroscience, University of Tübingen, Tübingen, Germany; e Institute for Modelling and Simulation of Biomechanical Systems, University of Stuttgart, Stuttgart, Germany

**Keywords:** Face, Neurophysiology, OPM, MMG, Palsy, Facial nerve disorders

## Abstract

**Objective:**

Measuring facial muscle activity is crucial in the diagnosis of facial palsy. This study investigated whether contactless Magnetomyography (MMG) using optically pumped magnetometers (OPM) is feasible for visualizing facial muscle activity.

**Methods:**

An anatomically shaped mask featuring eleven OPM was arranged on one side of the face of five healthy subjects. MMG was recorded while they performed different facial expressions. The root mean square of each OPM signal was calculated for each expression and subject and allocated to the individual face. Moreover, the maximum average muscle activity and the signal-to-noise ratio (SNR) were determined.

**Results:**

The subjects’ facial muscle activity could be measured individually per facial expression. Mean RMS was 0.6pT (SD 0.4pT), resulting in a mean SNR of 2.2 (SD 1.2).

**Conclusions:**

Imaging facial activity via MMG using OPM is possible, although the sensor positioning (sensor geometry and distance to the muscle) is decisive. However, the signal amplitude of the facial muscles is low and the interindividual anatomical variability renders the measurement setup challenging.

**Significance:**

As the imaging of facial MMG is feasible, this study paves the way for future studies using OPM for the diagnosis, monitoring, and rehabilitation of facial muscle and facial nerve disorders.

## Highlights

• Initial evidence that optically pumped magnetometers can non-invasively measure facial muscle activity via magnetomyography.
• Investigation of the amplitude characteristics of facial muscles.
• Proposal for technical improvements to facilitate facial magnetomyography use in future clinical diagnostics.

## 1 Introduction

Facial nerve palsy leads to both functional and aesthetic impairments as well as to psychological and social burdens (Li, et al., 2016, Cuenca-Martínez, et al., 2020). While the prognosis for recovery from idiopathic facial nerve palsy is considered favorable, non-idiopathic paresis is less likely to fully heal (Peitersen, 1982, Sullivan, et al., 2007, Engström, et al., 2008, Heckmann, 2023).

The measurement, quantification, and imaging of muscle activity in the face together represent a central component of the diagnosis of facial nerve palsy (Alford, 1967, Andresen, et al., 2020, Zaccor and Wang, 2022). Indeed, during the initial stage of flaccid facial nerve palsy, it can help to estimate the prognosis of recovery. Furthermore, synkinesia and autoparalytic coactivation of muscle groups can both be detected and therapeutically addressed during the post-paralytic stage with defective healing (Grosheva and Guntinas-Lichius, 2007). However, a purely visual description of the clinical findings of facial nerve palsy often lacks sufficient objectivity and reliability, despite the availability of highly developed scoring systems (Kanerva, Poussa and Pitkäranta, 2006). In this regard, automated photo and video analyses are promising tools that could soon be used in routine clinical practice. Still, their assessments are also based on the evaluation of muscle excursions and so do not adequately reflect the underlying neuromuscular pathology, especially in cases of chronic facial nerve palsy, where there is often a mixed picture comprised of hypo- and hyperactive muscle groups, synkinesia, and antagonizing coactivations of different muscles (Mothes et al., 2019).

Needle electromyography (EMG) is considered the gold standard for detecting voluntary muscle activity or pathological spontaneous activity (Urban et al., 2020). In addition, the examination can prove beneficial for assessing the prognosis of idiopathic facial nerve palsy (Petrides et al., 2023). The main disadvantage of needle electromyography, however, is the invasiveness of the examination, which can lead to pain and complications such as hematomas, bleeding, or infections. Another disadvantage is that only one locus can be measured at a time. This is problematic because facial expressions and pathological movement patterns are not generally caused by the activation of individual mimic muscles but by a complex interplay of different muscle groups. Hence, to measure the entirety of the facial muscle activity and detect pathological movement patterns, repetitive measurements of different muscles must be performed. Alternatively, a setup involving simultaneous recordings from a large number of electrodes can be used. However, the examination procedure may be too time-consuming for the clinical routine because many surface electrodes have to be applied to the patient’s face.

An alternative to EMG entails the contactless measurement of the magnetic counterpart to electromyography – namely, magnetomyography (MMG) (Elzenheimer, et al., 2020, Broser et al., 2021, Broser et al., 2021). The measurement of biomagnetism using conventional sensors (via a superconductive quantum interference device [SQUID]) is technically complex and geometrically inflexible due to the need for cryogenic cooling to a temperature of −269 °C. However, recent technical developments in the form of small-format, highly sensitive optically pumped magnetometers (OPM) open up the possibility of individual anatomical sensor positioning.

This study sought to investigate to what extent facial muscle activity can be measured and mapped using non-invasive MMG with OPM. For this purpose, we investigated the facial MMG of five healthy subjects that were performing different facial expressions.

## 2 Methods

### 2.1 Study population

Five healthy volunteers participated in this study (2 women and 3 men, mean age: 30.4 years, standard deviation: 3.5 years). All the study participants were authors and provided written consent to participate and to the publication of the research data, therefore ethics committee approval was not required. The examinations were conducted in August 2023 at the Department of Otorhinolaryngology, Head and Neck Surgery at the University Hospital of Tübingen. All test subjects wore uniform, non-ferromagnetically active clothing. One subject was known to wear a dental retainer; all the others confirmed that they had no metal in or on their bodies.

### 2.2 Study design

The five healthy volunteers were examined as a proof-of-feasibility study. We hypothesized that it is possible to measure the activity of mimic muscles using MMG and that different activity patterns will be detected depending on the facial expression performed.

### 2.3 Experimental setup

MMG was performed in supine position using an array of FieldLine v2 OPM (FieldLine, Boulder, CO, USA) in a magnetically shielded tube obtained from the same manufacturer. Using custom-made plaster masks, 11 OPM were placed over the right side of each subject’s face according to the Kuramoto scheme (Kuramoto et al., 2019). Care was taken to ensure that the distance between the skin and the sensor was as small as possible while avoiding contact between the skin and the sensor during facial movements. The fixed sensory geometry and movement of the face caused a relative movement of the signal source in relation to the sensors. In addition, a non-magnetic analog button connected to the measurement system was positioned in each subject’s right hand, which allowed the subject to indicate facial activity intervals and eye blinks.

### 2.4 Procedure

Each subject performed 11 blocks of 90 s duration. In each block one of eleven standardized facial expressions was performed (Schaede et al., 2017). During each block, the subjects repeatedly switched between executing the standardized facial expressions and rest (mean 21.3 repeats, SD = 3.3). The standardized facial expressions were as follows: 1. Wrinkle forehead; 2. Gentle closure of the eyes; 3. Firm closure of the eyes; 4. Wrinkle nose; 5. Smile with a closed mouth; 6. Smile with an open mouth; 7. Pucker lips; 8. Blow out the cheeks; 9. Show teeth; 10. Pull down the corners of the mouth; 11. Natural smile.

### 2.5 Analysis

**Fig. 1:**
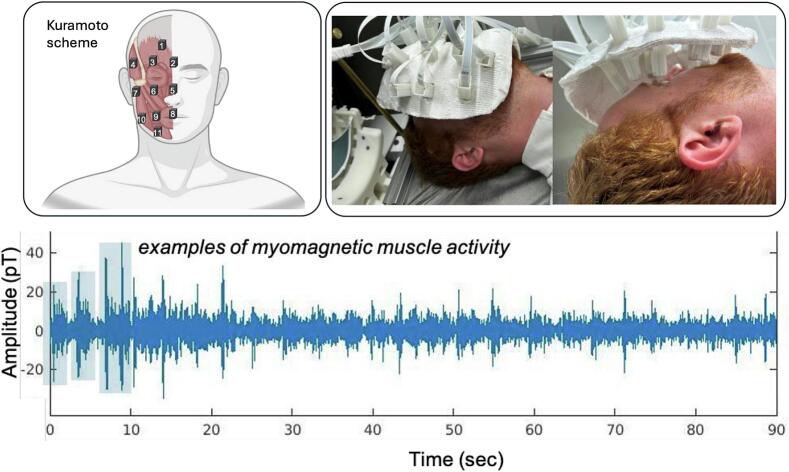
Experimental setup. Top left: Sensor positioning according to the Kuramoto scheme. Top right: Experimental setup. Bottom: Raw signal of myomagnetic muscle activity (exemplary).

The OPM signal was recorded orthogonally to the skin on the Z-axis at a sampling rate of 1000 Hz. The data were analyzed using MATLAB (version R2022b, MathWorks Inc., Natick, MA, USA) with custom scripts and the FieldTrip toolbox (Oostenveld *et al.*, 2010). Boxplots were created using JMP 16.0 (SAS Institute Inc., Cary, NC). Data analysis of the 12 recorded channels (11 OPMs and 1 trigger channel) included the following steps (Fig. 1): Based on previous MMG studies, the data were band-pass filtered between 30 and 90 Hz and with a 50 Hz notch filter (Marquetand et al., 2021). The sensor data were segmented into sections corresponding to the activity phases using the rectangular trigger signal. Signal artifacts were visually detected and corresponding sections were omitted. The root mean square (RMS) of signals was calculated for each segment. For the initial 90 s rest period, segments with eye blinks were excluded according to the trigger signal and RMS was computed in 1 s intervals. The rest RMS was defined as the background noise per OPM sensor and subject. From the RMS values of the individual segments, the mean for each sensor per trial (facial expression) and subject was calculated (mean RMS). These means were related to the individual RMS of the background noise for each sensor and subject to obtain the signal-to-noise ratio as a measure of activity for each sensor, trial (facial expression), and subject (mean signal-to-noise ratio [SNR]). To visualize the activity patterns, the measured sensor values were allocated to the corresponding sensor position in the Kuramoto diagram for each trial and subject. Furthermore, we examined RMS values at rest and of the standardized facial expressions “forced eyelid closure,” “showing teeth,” and “natural smile” inter-individually. Finally, an analysis of the distribution of the measured values across the face was carried out depending on the facial expression performed. This analysis provides the regional magnetic flux densities of the anatomical regions of the face depending on the facial expression. For this purpose, the sensors were segmented and analyzed separately depending on the facial movement performed and based on their position over the corresponding muscle areas (The analysis sequence is available in the Appendix, see Table 1).

**Table 1:** Analysis Sequence for mean RMS per anatomical region as a function of the facial expression performed.

Sequence	OPM-Channels	Mean RMS
1. Wrinkle forehead	1–4	0,4 (SD 0,1)
2. Gentle closure of the eyes	2–7	0,4 (SD 0,2)
3. Firm closure of the eyes	2–7	0,7 (SD 0,5)
4. Wrinkle nose	2, 3, 5, 6	0,6 (SD 0,4)
5. Smile with a closed mouth	5–11	0,6 (SD 0,3)
6. Smile with an open mouth	5–11	0,7 (SD 0,3)
7. Pucker lips	8–11	0,5 (SD 0,2)
8. Blow out the cheeks	5–11	0,7 (SD 0,4)
9. Show teeth	5–11	0,8 (SD 0,4)
10. Pull down the corners of the mouth	8–11	0,5 (SD 0,3)
11. Natural smile	5–11	0,7 (SD 0,4)

All statistical analyses were carried out using JMP version 16.0. The anonymized data can be made available upon reasonable request.

## 3 Results

**Fig. 2:**
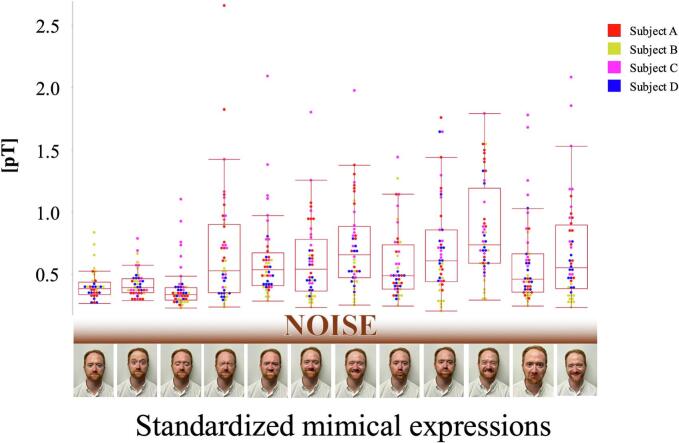
Mean RMS of every sensor per trial across all the subjects.

Muscular activity was successfully recorded by means of MMG in four out of five subjects. The fifth subject wore an enoral wire (retainer) made of a ferromagnetic alloy on the upper teeth, which led to artifacts that made it impossible to measure magnetic muscle activity. Fig. 2 illustrates the measured mean RMS across all sensors, subjects and facial expressions.

The background noise (rest condition) ranged between 0.25 and 0.33pT across all subjects. The facial expression with the lowest amplitude was “relaxed eyelid closure” with a mean RMS of 0.42pT and corresponding mean SNR of 1.48. By contrast, an average field strength of 0.68pT was measured for “forced eyelid closure”. The highest average amplitudes were achieved with the facial expression “show teeth” with a mean RMS of 0.85pT and a mean SNR of 2.98. The highest individual values (averaged over a measurement sequence of 90 s) were measured at 2.66pT (“firm closure of the eyes,” sensor position 2, subject A). Occasionally, magnetic flux densities of more than 4pT occurred during the “wrinkle forehead” (4.35pT) and “firm closure of the eyes” (4.30pT) facial expressions (over a two-second measurement sequence), which resulted in a maximum SNR of 14.18 and 16.65, respectively. Table 2 summarizes the results of the RMS and SNR across all subjects and sensors.

**Table 2:** Mean RMS and SNR across all sensors (4 subjects with 11 sensors each, n = 44) per facial expression performed (mean RMS in pT).

	R	WF	GCE	FCE	WN	SCM	SOM	PL	BC	ST	PCM	NS
Mean RMS(SD)	0.29 (0.05)	0.42 (0.11)	0.39 (0.18)	0.68 (0.46)	0.62 (0.33)	0.61 (0.32)	0.72 (0.36)	0.58 (0.28)	0.71 (0.40)	0.85 (0.39)	0.59 (0.35)	0.68 (0.41)
Mean SNR (SD)	−	1.48 (0.30)	1.34 (0.45)	2.43 (1.71)	2.15 (1.03)	2.11 (1.03)	2.53 (1.21)	2.03 (0.96)	2.40 (1.15)	2.98 (1.31)	2.01 (0.94)	2.33 (1.25)

Note: R = Rest (i.e., background noise), WF = Wrinkle forehead, GEC = Gentle closure of the eyes, FCE = Firm closure of the eyes, WN = Wrinkle nose, SCM = Smile with closed mouth, SOM = Smile with opened mouth, PL = Pucker lips, BC = Blow out cheeks, ST = Show teeth, PCM = Pull down corner of the mouth, NS = Natural smile.

**Fig. 3:**
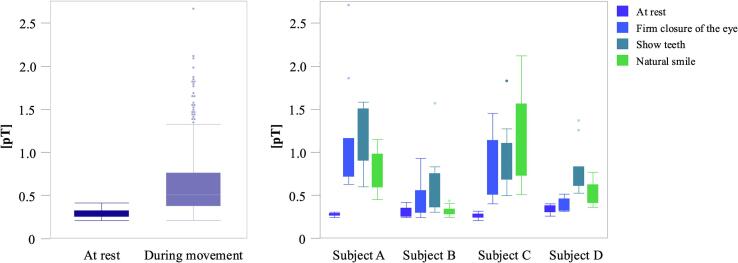
Mean RMS at rest vs. during movement across all the subjects (left) and the mean RMS during selected facial expressions per subject (right).

The measurements across all the sensors and subjects revealed a significant difference between “rest” and “movement” in terms of the mean RMS (at rest: 0.29 ± 0.05 (SD), during movement: 0.62 ± 0.36 (SD); T-test: t = 18.7; p < 0.0001) (see Fig. 3). There were significant intra-individual differences between the subjects for the measurements taken at rest and during activity for the “Firm closure of the eyes,” “showing teeth,” and “natural smile” facial expressions (see Table 3; ANOVA, F [3,40] = 8.995, p < 0.0001).

**Table 3:** Mean RMS at rest and during selected movements.

	R	FCE	ST	NS	ANOVA
Mean RMS (SD) Subject A	0.27 (0.02)	1.09 (0.63)	1.15 (0.36)	0.77 (0.24)	*p* < 0.0001
Mean RMS (SD) Subject B	0.30 (0.05)	0.45 (0.20)	0.61 (0.36)	0.31 (0.05)	*p* = 0.003
Mean RMS (SD) Subject C	0.25 (0.03)	0.83 (0.32)	0.89 (0.37)	1.12 (0.52)	*p* < 0.0001
Mean RMS (SD) Subject D	0.33 (0.05)	0.37 (0.07)	0.77 (0.27)	0.52 (0.12)	*p* < 0.0001

**Fig. 4:**
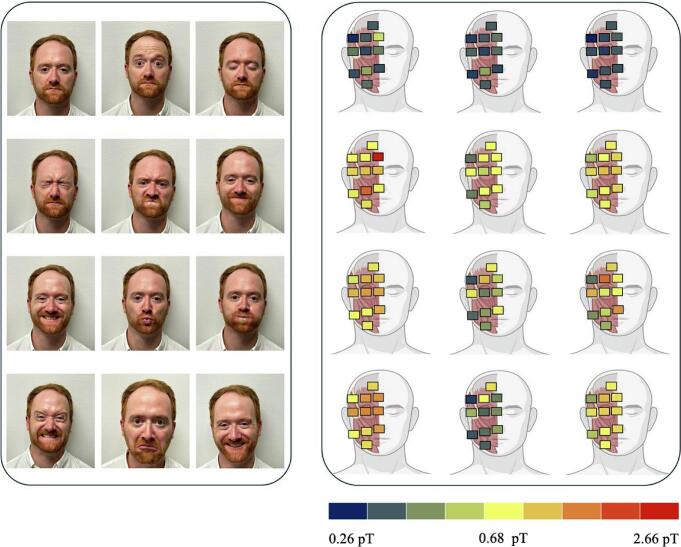
Scalograms of the mean RMS per facial expression. Left: Performed facial expressions. Right: Distribution of the RMS per facial expression performed; min–max distribution equally across all the facial expressions (absolute values are provided in the Appendix, see Table 4).

The following scalogram show and visualize the average RMS of a test person at rest and for the facial expressions corresponding to the images on the left (Fig. 4).

**Fig. 5:**
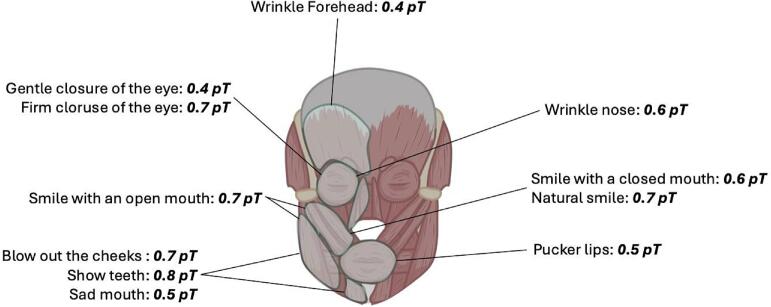
Mean RMS per anatomical region as a function of the facial expression performed.

Fig. 5 shows movement-specific, regional magnetic flux densities. For this purpose, the sensors were segmented and analyzed depending on the facial movement performed and their position over the corresponding muscle areas. The mean RMS rose with increasing intensity of the performed movement by the test subject. For example, the mean RMS in the periocular region for the “gentle closure of the eye” expression was 0.4pT, whereas a mean RMS of 0.7pT was measured over the magnetically active muscle regions for the “firm closure of the eye” expression. The highest mean RMS of 0.8pT was achieved in the perioral muscle region for the “Show Teeth” expression.

## 4 Discussion

In this proof-of-feasibility study, the results in four healthy subjects revealed that it is possible to measure facial muscle activity using magnetomyography with optically pumped magnetometers. To the best of our knowledge, this study is the first to use an anatomically oriented arrangement of OPM to measure activity patterns of facial muscles.

The measurements of the magnetic flux densities at rest and during active facial movement differed significantly, thereby demonstrating the measurability of the movements of the mimic musculature using OPM. The mean magnetic field strength with relaxed facial muscles was 0.29pT (SD = 0.05pT). Voluntary facial movements more than doubled the average signal, with measurements on average yielding values of about 0.62pT and an SNR of about 2.14. The measured values differed both inter-individually and intra-individually depending on the facial expression. Simultaneous measurement at several positions on one side of the face made it possible to visualize the facial muscle activity with different activity patterns depending on the facial movement performed. However, it must be noted that the spatial resolution of the measurement set-up used in this study is not sufficient to draw conclusions about the facial expressions performed based on the scalograms alone. But measuring and analyzing the magnetic flux densities specifically according to facial expression over the corresponding muscularly active anatomical region showed that the mean magnetic flux densities differed depending on the anatomical region and rise with increasing intensity of the movement (gentle closing of the eye vs. firm closing of the eye).

The gold standard for assessing facial muscle activity is EMG and visual assessment using photo and video analysis as described in the introduction. Its advantages lie in the method’s ubiquitous availability, ease of use, and high sensitivity to pathological changes. Invasive needle EMG, which has a high spatial resolution, and EMG using surface electrodes are regularly used. In both cases, however, direct contact must be established and maintained between the measuring instrument and the muscle to be measured. It is challenging to use Needle EMG in children due to its invasiveness and painfulness. In routine clinical practice, only a selection of the mimic muscles is usually measured, as electromyographic imaging of all the facial muscles would require repeated skin perforations by the EMG needle or the application of a large number of surface electrodes. Yet, measuring only individual mimic muscles does not provide an adequate picture of the overall activity of the face, given that movements of the face require the coordinated co-activation and detonation of different muscle groups.

In light of the disadvantages of needle-electromyography Fridlund and Cacioppo, 1986, Kuramoto, et al., 2019, and Mueller et al. (2022) presented arrangements of multiple simultaneously conducting surface electrodes to accurately map global patterns of the facial muscle activity in the context of various standardized movements. For instance, Mueller et al. (2022) compared two different electrode arrangements using the Fridlund and Kuramoto schemes, concluding that a symmetrical arrangement of the electrodes across the entire face is superior to a purely topographically oriented arrangement in terms of detecting specific muscle activity patterns. High-density surface EMG for measuring mimic muscle activity has not yet been adopted for routine clinical use, likely due to the considerable time required for its complex setup. Additional limitations include challenges in application for individuals with beards, makeup, severely seborrheic skin, or certain dermatological conditions and sensitivities.

Magnetomyography, the measurement of the magnetic fields generated by the skeletal muscles, was described over 50 years ago and presented as a possible alternative to EMG (Cohen and Givler, 1972). The rapidly advancing developments in the field of quantum sensor technology form the basis of the experimental setup used in the present study. The use of OPM, which contrast with magnetic field measurement using SQUID sensors by not requiring cooling, represents a significantly more cost-efficient approach and allows a far more flexible sensor arrangement over the object to be measured. This renders the anatomical distribution over the face according to the Kuramoto scheme possible. Experience of the MMG technique is limited, although recent studies have shown numerous potential applications. In particular, patients with neuromuscular diseases who have been dependent on regular and sometimes painful needle EMG examinations could benefit from the further development of this method (Ghahremani Arekhloo et al., 2023).

To date, the measurement of magnetic potentials has been technically complex, which is supported by the results of this study. The low signal strength in the pico- or femtotesla range when compared with EMG, the lower SNR, and the complex and expensive examination conditions regarding ferromagnetic shielding make MMG challenging (Zuo, et al., 2020, Ghahremani Arekhloo, 2023). In addition, the measurement of pathological spontaneous activity is only possible to a minimal extent with MMG due to the low SNR. Fibrillations could only be detected in the significantly more voluminous skeletal muscles (Marquetand et al., 2021). The unique characteristics of the mimic musculature further complicate measurement using MMG in the face. When compared with the skeletal muscles, the mimic muscles have a minimal volume. The orbicularis oris muscle, for instance, has a volume of only 80–320 mm^3^, while the depressor anguli oris muscle has a volume of around 420–1140 mm^3^ (Volk et al., 2014). This also leads to a low signal amplitude and SNR when compared with the skeletal muscles, where Amplitudes have been shown to reach values above 10pT (Nordenström et al., 2024). The mimic musculature is a complex, three-dimensional network of muscle fibers with different directions of course and pull. However, the measurement of magnetic currents is vector-based, and the interpretation is more complicated if the direction of the muscle fibers is indeterminate (Broser, et al. (2021)). Furthermore, facial movements cause relative movements between the mimic muscle to be captured and the fixed sensor located above the face, which means that the object to be measured can change during the measurement process. A further limitation is that MMG is not applicable for people who carry ferromagnetic metal in or on their body due to artefact build-up.

The advantages of MMG lie in the non-contact nature of the measurement, which avoids the painful insertion of needles. However, a technical limitation is that the current technology does not appear to be able to detect pathological spontaneous activity of the mimic musculature. Another benefit is that the magnetic field is less influenced by the tissue between the muscle and the sensor (e.g., skin, subcutaneous fatty tissue) than is the case for the electrical potential in surface EMG (Broser et al., 2021, Broser et al., 2021). Facial MMG also has the potential to contribute to the more comprehensive detection of even complex movement disorders of the face. Simultaneous recording via several sensors that are evenly distributed across the face can contribute to detecting hypo- and hyperactive areas, as well as synkinetically active areas, for instance, in the context of defect healing after an initial flaccid facial nerve palsy. This would allow botulinum toxin injections, for example, to be applied intramuscularly in a more targeted manner (Guzmán-Venegas, Araneda and Silvestre, 2014). In particular, in partially recovered facial nerve palsies with relevant defective healing, the value of surgical measures in relation to facial reanimation or synkinesia treatment remains unclear (Shokri et al., 2021). In these cases, a global view of the activity of the facial muscles appears to be mandatory, indicating that the potential benefit of facial MMG is substantial.

The present study’s findings showed that special attention must be paid to the sensor positioning. The greater the distance between the sensor and the target muscle, the weaker the signal strength (Garcia and Baffa, 2015). However, accidental contact with the sensor must be avoided to prevent artifacts. Therefore, this study used custom-made plaster masks that enabled sensor positioning according to the Kuramoto scheme. However, production of the masks was time-consuming and not feasible for routine clinical practice. Despite being individually adjusted, the sensor positioning was particularly complicated in the cheek and mouth area, as overly close positioning led to skin contact during specific movements (“cheeks inflate,” “pucker lips”) and thus required a somewhat more distant arrangement than in the forehead area (see Fig. 2). In the future, standardized OPM mounts, such as those incorporating a chin and forehead rest, could facilitate controlled head positioning and allow for precise, time-efficient adjustment of sensors to individual anatomy, thereby simplifying the examination procedure. In the experimental setup presented, uniaxial Fieldline v2 OPMs were utilized. Incorporating multiaxial OPMs could provide a valuable spatial dimension. A previous study demonstrated that triaxial MMG measurements effectively capture the pennation angle of muscle fibers, representing their three-dimensional orientation relative to tendons (Broser et al., 2021, Broser et al., 2021). Employing an array of multiaxial OPMs for facial MMG could enhance spatial resolution for mapping facial muscle fiber directions. However, the small size of facial muscles may pose challenges for such analyses. Nonetheless, this study demonstrates the feasibility of facial MMG using uniaxial Fieldline OPMs, laying the groundwork for more complex experimental setups in the future. In sum, due to the abovementioned prerequisites, measuring facial MMG is challenging and requires highly developed OPM measurement and analyzing technology and optimal sensor positioning.

## 5 Conclusion

In conclusion, the present study suggests that MMG for imaging facial muscle activity is feasible. Further studies are required to optimize the recording setup, increase spatial resolution, to compare healthy subjects and patients and to assess the clinical potential of facial MMG for a better understanding of the pathophysiology of facial nerve palsies and for improving treatment outcomes. The technical progress of the last 50 years fosters optimism that further developments in the field of magnetic sensor technology and information technology will make it possible to overcome the existing technical limitations with the aim of using MMG in everyday clinical practice as a diagnostic tool for neuromuscular diseases.

## Figures and Tables

**Table 4:** Mean rms in pt per sensor and facial expression for one exemplary subject.

Sensor	R	WF	GCE	FCE	WN	SCM	SOM	PL	BC	ST	PCM	NS
1	0,34	0,34	0,33	0,61	0,61	0,62	0,80	0,75	0,97	0,94	0,86	0,73
2	0,51	0,38	0,35	2,66	0,62	0,94	1,07	0,49	0,72	1,47	0,39	0,84
3	0,34	0,34	0,31	0,70	0,72	0,57	1,17	1,14	1,76	1,42	0,61	1,13
4	0,27	0,30	0,26	0,62	0,37	0,47	0,52	0,33	0,37	0,59	0,29	0,44
5	0,39	0,36	0,34	1,14	0,56	1,04	1,30	0,46	0,55	1,50	0,40	0,95
6	0,31	0,31	0,30	1,06	0,49	1,07	1,38	0,39	0,53	1,55	0,35	0,99
7	0,38	0,35	0,32	0,98	0,68	0,94	1,17	0,79	1,19	1,40	0,46	0,86
8	0,32	0,31	0,30	0,72	0,62	0,95	1,20	0,69	1,44	1,32	0,43	0,91
9	0,44	0,44	0,33	1,82	0,55	0,65	0,69	0,43	0,50	0,89	0,36	0,58
10	0,32	0,30	0,28	0,75	0,39	0,51	0,56	0,36	0,41	0,62	0,32	0,48
11	0,36	0,34	0,32	0,88	0,48	0,69	0,78	0,42	0,45	0,88	0,35	0,61

## Appendix

See Table 1.

See Table 4.
